# Circulating tumor cell-derived exosome–transmitted long non-coding RNA TTN-AS1 can promote the proliferation and migration of cholangiocarcinoma cells

**DOI:** 10.1186/s12951-024-02459-8

**Published:** 2024-04-18

**Authors:** Xu Zhou, Xiaohan Kong, Jun Lu, Heng Wang, Meng Liu, Shuchao Zhao, Zhaozhi Xia, Qinggong Liu, Hongrui Sun, Xin Gao, Chaoqun Ma, Zheyu Niu, Faji Yang, Xie Song, Hengjun Gao, Shizhe Zhang, Huaqiang Zhu

**Affiliations:** 1 Department of Hepatobiliary Surgery, Shandong Provincial Hospital Affiliated to Shandong First Medical University, No. 324, Jingwu Road, Jinan, Shandong 250021 China; 2 Department of Hepatobiliary Surgery, Cheeloo College of Medicine, Shandong Provincial Hospital, Shandong University, Jinan, Shandong 250021 China

**Keywords:** Cholangiocarcinoma, Circulating tumor cells, Exosome, Titin-antisense RNA1, Metastasis

## Abstract

**Background:**

Exosomes assume a pivotal role as essential mediators of intercellular communication within tumor microenvironments. Within this context, long noncoding RNAs (LncRNAs) have been observed to be preferentially sorted into exosomes, thus exerting regulatory control over the initiation and progression of cancer through diverse mechanisms.

**Results:**

Exosomes were successfully isolated from cholangiocarcinoma (CCA) CTCs organoid and healthy human serum. Notably, the LncRNA titin-antisense RNA1 (TTN-AS1) exhibited a conspicuous up-regulation within CCA CTCs organoid derived exosomes. Furthermore, a significant elevation of TTN-AS1 expression was observed in tumor tissues, as well as in blood and serum exosomes from patients afflicted with CCA. Importantly, this hightened TTN-AS1 expression in serum exosomes of CCA patients manifested a strong correlation with both lymph node metastasis and TNM staging. Remarkably, both CCA CTCs organoid-derived exosomes and CCA cells-derived exosomes featuring pronounced TTN-AS1 expression demonstrated the capability to the proliferation and migratory potential of CCA cells. Validation of these outcomes was conducted in vivo experiments.

**Conclusions:**

In conclusion, our study elucidating that CCA CTCs-derived exosomes possess the capacity to bolster the metastasis tendencies of CCA cells by transporting TTN-AS1. These observations underscore the potential of TTN-AS1 within CTCs-derived exosomes to serve as a promising biomarker for the diagnosis and therapeutic management of CCA.

**Supplementary Information:**

The online version contains supplementary material available at 10.1186/s12951-024-02459-8.

## Background

Cholangiocarcinoma (CCA) is the most common and aggressive malignancy of the bile ducts. It exhibits high heterogeneity, malignancy, and complexity in signaling pathways, leading to poor prognosis and a propensity for metastasis [1, 2]. Due to the inconspicuous early symptoms, lack of specific molecular markers, and suitable early diagnostic methods, the majority of CCA patients are diagnosed at advanced stages, resulting in a less than 10% 5-year survival rate [3, 4]. Consequently, there is an urgent need for new biomarkers and therapeutic targets to enhance early detection and patient prognosis. Simultaneously, an in-depth exploration of the tumor microenvironment is essential to uncover the mechanisms underlying the progression and metastasis of cholangiocarcinoma.

Circulating tumor cells (CTCs) refer to tumor cells that detach from solid tumors or metastatic sites and enter the peripheral bloodstream. They are a major factor contributing to postoperative recurrence and metastasis [5]. CTCs carry a wealth of molecular information from tumor cells, and downstream analyses of CTCs (such as in vitro culture and genetic sequencing) hold promising applications [6]. Constructing organoid models from CTCs cell lines for human-derived tumor studies holds significant importance in revealing the mechanisms behind tumor metastasis [7, 8]. Tumor-derived exosomes (TEX), originating from tumor cells, are membranous vesicles that carry biologically active substances such as proteins and nucleic acids. Constituting a component of the tumor microenvironment, TEX play a pivotal role in mediating intercellular communication and material exchange, thus playing a crucial role in tumor metastasis [9, 10]. Studies indicate that only a small subset of CTCs possess colonization and metastatic potential [11]. However, TEX can alter the extracellular matrix composition of tumor cells, induce epithelial-mesenchymal transition (EMT), thereby enhancing tumor cell invasiveness. TEX can also induce the formation of pre-metastatic niches in target organs, and after CTCs colonization, they can promote angiogenesis to facilitate tumor metastasis [12, 13]. Thus, TEX plays a significant role throughout various stages of tumor metastasis. Through TEX, CTCs are able to exchange information among tumor cells with distinct metastatic potential (Fig. 8B). Gaining insight into the mechanisms of TEX in the process of tumor metastasis and identifying key targets of TEX action will contribute to achieving targeted therapies for tumors and improving patient prognosis.

Researches have revealed that exosomes can protect long non-coding RNAs (LncRNAs), enabling their stable presence within the bloodstream [14]. The expression levels of exosomal LncRNAs can aid in distinguishing and diagnosing benign and malignant diseases [15]. Exosome-mediated LncRNAs play a critical role in tumor resistance to therapies [16]. Multiple lines of evidence suggest that exosomes are implicated in various aspects of tumor development, growth, metastasis, and drug resistance, with TEX (tumor-derived exosomes) being particularly notable [17–19]. In this study, for the first time, we conducted transcriptome sequencing on exosomes secreted by cholangiocarcinoma (CCA) CTCs, delving deep into the tumor microenvironment of CCA. The primary objective is to explore the potential of specific LncRNA molecules within CCA CTC-derived exosomes as potential markers for CCA, investigating whether they are involved in regulating the biological functions of CCA. This endeavor opens up new avenues for research in the diagnosis and treatment of CCA.

## Methods

### Sample collection and ethics

This study was approved by the ethics committee of our hospital (No. 2021 − 553; China Clinical Trial Registration Center-Registration No. ChiCTR2300072761), and informed consent was obtained from participants in accordance with respective committee regulations. Samples were collected from 32 cases of cholangiocarcinoma (CCA) patients who received treatment at our institution from June 2018 to January 2023. The samples encompassed 15 mL of blood, tumor tissues, and normal tissue specimens located 5 cm away from the tumor tissue edge for each CCA patient. Tissue specimens were promptly frozen and preserved in liquid nitrogen, while blood samples were stored and transported at 4 °C and processed within 72 h. Additionally, blood samples of 8 mL were collected from 32 healthy individuals undergoing routine health check-ups.

### Material and instrument

Human cholangiocarcinoma cell lines (Huh28, CCLP1, TFK-1, SK-ChA-1, FRH-0201, KMBC, QBC939) and normal human intrahepatic bile duct epithelial cells (HIBEC) were obtained from Cell Bank/Stem Cell Bank, Chinese Academy of Science in Shanghai. Culture media, serum, and trypsin were procured from Gibco. Organoid culture media [20–22] and cryopreservation solution were acquired from Shanghai JiaYuan Bio-Technology Co., Ltd. qRT-PCR detection kits, miRNeasy Mini kits (Qiagen), and Trizol reagent were sourced from Beyotime Biotechnology Co., Ltd. Antibodies including CD63, CD9, CD81, Calnexin, Ki67, Cyclin D1, E-cadherin, Snail, N-cadherin, Slug, ZEB1, Twist, and GAPDH were purchased from Abcam. Magnetic separation rack, Fe_3_O_4_, cell filters, dimethyl dioctadecyl ammonium chloride (GHDC), hexadecyl trimethyl ammonium chitosan chloride (HQCMC), and 1,2-dioleoyl-sn-glycero-3-phosphocholine (DOPC) were purchased from LieYuan (Shanghai) Biomedical Technology Co., Ltd. Cholesterol (Chol) was sourced from Shanghai YuanYe Bio-Technology Co., Ltd. N-hydroxysuccinimide (NHS) and 1-ethyl-3-(3-dimethylaminopropyl)-carbodiimide (EDC) were procured from Morey (Shanghai) Bioscience Co., Ltd. Dichloromethane and other common reagents were obtained from China National Pharmaceutical Group Corporation. Integra-ted vacuum concentrator was purchased from Shanghai Bannuo Biotechnology Co., Ltd (BIONOON VAC-AIO).

### Organoid culture of CTCs

7 mL of blood was added to a cell filtration tube and centrifuged for 3 min at 1000 rpm/min. After centrifugation, the cell filtration membrane was washed with 3 mL of PBS and centrifuged for an additional 3 min. The cells on the filtration membrane were then washed with 1 mL of PBS and transferred to a centrifuge tube and washed for three times. Following by another time of centrifugation of 3 min and removal of the supernatant. After adding 1 mL of cooled PBS, centrifugation was performed for 3 min, and the supernatant was discarded. The cell pellet was resuspended in 50 µL of matrix gel on ice and then transferred to a 96-well plate. The plate was incubated in a cell culture incubator for 5 min. Subsequently, 100µL of organoid culture medium was added to each well, and the culture dishes were placed in the incubator under conditions of 37 °C and 5% CO_2_. The culture medium was replaced every 3 days. Cell growth was observed every 7 days, and CTCs organoids would be successfully cultured after 35 days of incubation. For cell staining, 10 µL of CK8/18/19-FITC, DAPI, and CD45-PE were added separately. The staining was performed in the dark for 20 min, followed by centrifugation to collect the cell pellet. Then, 20 µL of PBS was added to the cell pellet for mixing, and the cell suspension was dropped onto a glass slide. CTCs were identified using a fluorescence microscope [7, 8].

### Preparation and characterization of magnetic spheres

DOPC (1000 mL, 5 mg/mL), Cholesterol (6 mg), and HQCMC (1000 mL, 5 mg/mL) were dissolved in chloroform. Subsequently, 1 mL of Fe_3_O_4_ (50 mg/mL) and 6 mL of distilled water were added. Emulsification was carried out using a probe-type ultrasonic instrument with a power setting of 27%. Each ultrasound cycle consisted of 2 s of ultrasound followed by 1 s of pause, with a total reaction time of 6 min at a temperature of 25 °C. After rotary evaporation at room temperature for 30 min, lipid magnetic spheres (LMS) were obtained. GHDC (0.1 mg) was dissolved in 1 mL of isopropanol, NHS (0.1 mg) and EDC (0.1 mg) coupling agents were added separately. At the same time, 60 µg of CD63 antibody was added, and the mixture was co-incubated for 24 h to obtain CD63-GHDC. CD63-GHDC was then added to 1 mL of LMS, and the solution was vortexed every hour. After continuous reaction for 24 h, the solution of CD63-modified LMS (CD63-LMS) was obtained. CD63-LMS was subjected to magnetic separation for 10 min to remove the solution and eliminate unbound antibodies. Next, 2 mL of PBS was added to the obtained CD63-LMS, and the solution was stored at 4 °C [23, 24].

### CD63-LMS characterization test

After freeze-drying CD63-LMS, the magnetic properties were assessed using a vibrating sample magnetometer (VSM). The particle size and potential distribution of the CD63-LMS solution were measured using a particle size analyzer. The morphology of CD63-LMS was observed using atomic force microscopy (AFM) and transmission electron microscopy (TEM). UV-visible spectroscopy was employed for UV spectra analysis. Fourier-transform infrared spectroscopy (FTIR) was conducted for infrared spectral analysis.

### Isolation and identification of exosomes by CD63-LMS

Add 20 µL CD63-LMS to 2 mL of serum or cell suspension and incubate at room temperature for 30 min. Perform magnetic separation for 10 min and remove the supernatant. Wash with 2 mL of PBS solution three times to obtain CD63-LMS-captured exosomes. Then, add 2 mL of trypsin for digestion for 10 min, followed by vortexing with 2 mL of trypsin inhibitor for 5 min. Immediately place the exosome solution on a magnetic separation rack and let it stand for 10 min to remove the CD63-LMS solution. Collect the supernatant to obtain the purified exosome solution. Particle size and potential of the exosomes were analyzed using a particle size analyzer, and the morphology was observed using TEM. Exosomal protein content was quantified using a BCA protein assay kit (Pierce), and the presence of CD9, CD81, calnexin were assessed by western blot. Exosomes were labeled with CM-Dil (Invitrogen) dye, incubated at 37 °C for 30 min, diluted with PBS to 10 mL, centrifuged at 1000 g for 30 min, and the labeled exosome solution was added to Huh28 cells. Cells were incubated at 37 °C for 24 h, discard the medium and wash twice with PBS. Finally, subjected to imaging analysis on a flow cytometer.

### LncRNA analysis of tumor-derived exosomes

Three successfully constructed cholangiocarcinoma CTC-derived organoid models were selected, along with three blood samples from healthy individuals. CD63-LMS was used to capture cholangiocarcinoma CTC-derived exosomes and healthy human serum exosomes separately. After extracting total exosomal RNA, library quality assessment was performed using the Agilent Bioanalyzer 2100, followed by sequencing on the Illumina Hiseq platform. Cuffcompare software, in conjunction with authoritative databases such as Ensembl, Gencode, and UCSC, was utilized to screen for LncRNA. The GOSeq tool (v1.34.1) was employed to identify significant Gene Ontology (GO) terms, with enrichment genes having a significance level below 0.05. An internal script was employed to enrich important differentially expressed genes within the Kyoto Encyclopedia of Genes and Genomes (KEGG) pathways.

### RNA extraction and RT-PCR

Cholangiocarcinoma cell lines were cultured in RPMI 1640 or DMEM medium at 37 °C under 5% CO_2_. Passaging was performed when the cell density reached 80%. Total RNA was separately extracted from cells, tissues, blood, and serum exosomes. According to the kit instructions, the required reverse transcription system was prepared, and cDNA was synthesized using a regular PCR instrument and stored at -80 °C for future use. The RT-PCR system consisted of 10 µL 2×Mixture, 0.3 µL upstream and downstream primers (10 µmol/L, as provided in Supplementary Table S1), 2 µL template cDNA, and 7.9 µL RNase-free water. The mixture was briefly centrifuged, and RT-PCR was performed on a Bio-Rad qPCR instrument. The thermal cycling protocol included an initial denaturation step at 94 °C for 10 min, followed by 45 cycles of amplification at 94 °C for 30 s, 55 °C for 30 s, and 72 °C for 30 s. The housekeeping gene GAPDH was used as an internal reference, and the 2^−ΔΔCt^ method was employed for relative quantification and analysis of the results.

### Effect of cholangiocarcinoma cell-derived exosome TTN-AS1 on the proliferation and migration of cholangiocarcinoma cells

Stable transfection cell lines with TTN-AS1 knockout and overexpression were established. Cell viability, colony formation, transwell migration, cell cycle, apoptosis, scratch assay, qRT-PCR, Western blotting, TTN-AS1 expression vector, and plasmid construction were examined (Table S2). Detailed descriptions of these methods can be found in the supplementary information [25].

### Animal experiment

This study was approved by the Animal Ethics Committee of the research institution (License No: SYXK20020009). Huh28 cell lines (1 × 10^7^ cells in 0.1 mL PBS) were co-cultured with exosomes secreted by cells from different groups and then subcutaneously injected into the right abdomen of BALB/c female nude mice (5 weeks old, 6 mice per group). Tumor growth was monitored every two days. Mice were euthanized two weeks later, tumor size and weight were then measured. Representative regions were selected using hematoxylin and eosin (H&E) staining. Anti-Ki67 antibody (ab238020, Abcam) was used for immunohistochemistry (IHC) [25].

### Statistical analysis

Statistical analyses were conducted using SPSS 23.0. Differences were compared using t-tests or one-way analysis of variance (ANOVA). The correlation between TTN-AS1 expression and clinical information was assessed using the chi-square test. ROC curve analysis was performed to evaluate the clinical diagnostic value. A significance level of *P* < 0.05 was considered statistically significant. Statistical significance was denoted as * for *P* < 0.05, ** for *P* < 0.01, and *** for *P* < 0.001.

## Results

### Successfully cultured CCA CTCs organoid cell line

**Fig. 1:**
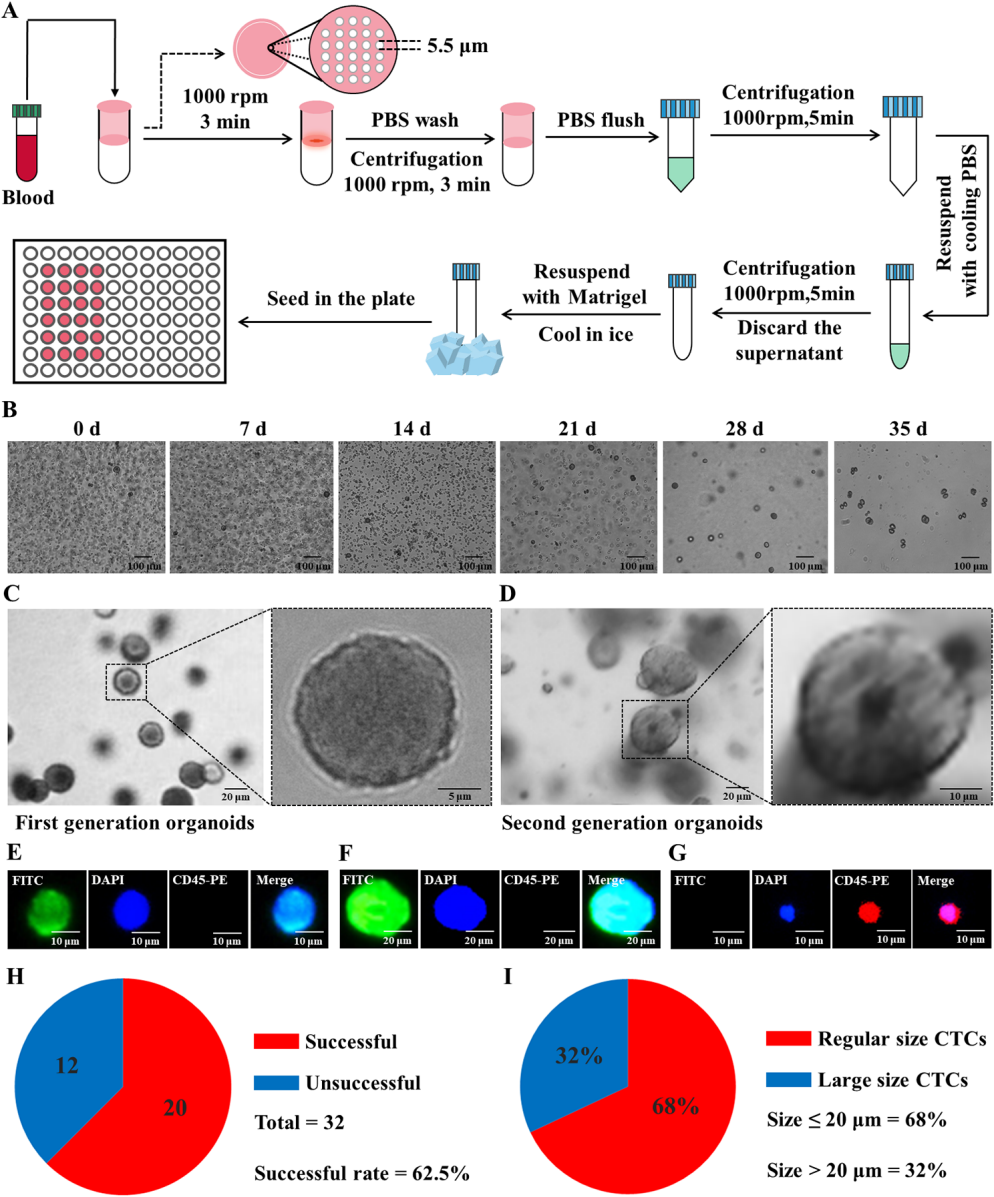
CTCs Organoid Culture Procedure and Results. (**A**) Workflow for CTCs isolation and culture with a pore size of 5.5 μm; (**B**) Growth status of CTCs; (**C**) Primary CTCs organoid culture at week 5; (**D**) CTCs organoid cell lines after passage; (**E**) Identification of normal-sized CTCs, diameter ≤ 20 μm, positive for DAPI and FITC, negative for CD45-PE; (**F**). Identification of large-sized CTCs, diameter > 20 μm, positive for DAPI and FITC, negative for CD45-PE; (**G**). Leukocyte identification, positive for DAPI and CD45-PE, negative for FITC; (**H**). CTCs culture outcomes, out of 32 collected cholangiocarcinoma samples, CTCs were successfully cultured in 20 samples, with a success rate of 62.5%; (**I**). Proportion statistics of normal-sized and large-sized CTCs

We successfully isolated and cultured CTCs from the blood of 32 CCA patients. The cell filter tube used consists of a filter and a round-bottom collection tube, with a microfiltration membrane sealing the bottom of the filter, featuring a pore diameter of 5.5 μm (Fig. 1A). Following CTCs isolation, they were cultured in organoid culture medium and placed in an incubator, with photographic documentation of organoid growth recorded every week (Fig. 1B). The successfully established first-generation CTCs organoid models exhibited heterogeneous near-spherical growth after 35 days (Fig. 1C). Upon passaging of the constructed CTCs organoids, cells exhibited irregular near-spherical shapes, with an obvious increase in cell volume for some cells (Fig. 1D). Immunofluorescence staining was used for the identification of the constructed CTCs organoid models (Supplementary Figure S1), categorizing CTCs into normal-sized CTCs (Fig. 1E, size ≤ 20 μm) and large-sized CTCs (Fig. 1F, size > 20 μm). Immunofluorescence staining was also employed to exclude non-tumor cells (Fig. 1G). Ultimately, we successfully isolated and cultured 20 CTCs organoid models from the blood of 32 CCA patients, achieving a survival rate of 62.5% for CTCs organoid construction (Fig. 1H). Among them, the proportion of normal-sized CTCs in the organoid models was 68%, while the proportion of large-sized CTCs was 32% (Fig. 1I).

### LMS characterization test analysis

**Fig. 2:**
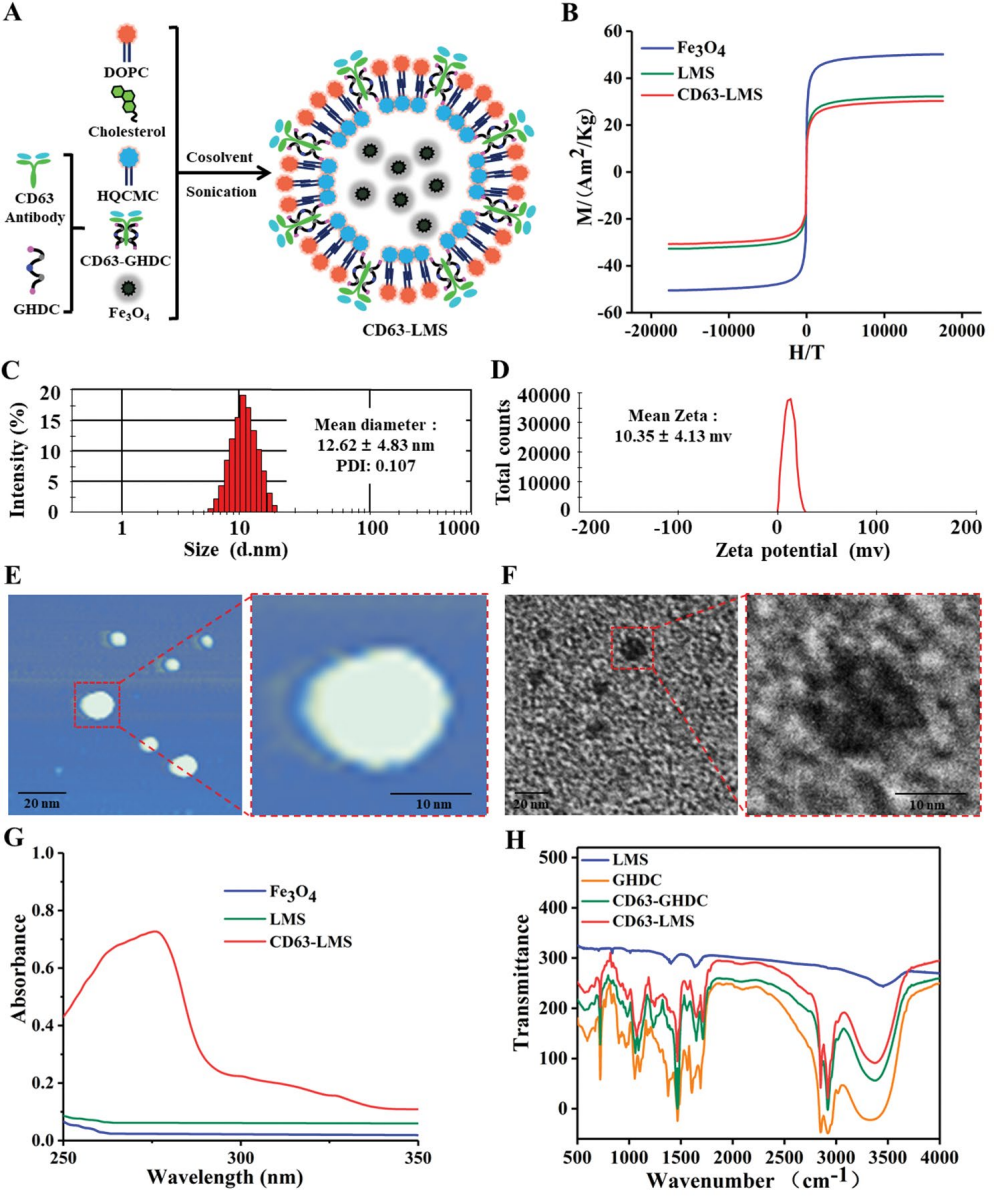
Preparation of Materials and Characterization Testing of CD63-LMS. (**A**) Flowchart of the preparation process; (**B**) Magnetic crystalline performance graph; (**C**) Particle size distribution; (**D**) Zeta potential distribution; (**E**) AFM observation; (**F**) TEM observation; (**G**) UV testing; (**H**) Infrared testing

According to the preparation procedure, CD63-LMS was obtained (Fig. 2A). The saturation magnetization of the prepared CD63-LMS, LMS, and Fe_3_O_4_ were 30.28 Am^2^/kg, 32.27 Am^2^/kg, and 50.19 Am^2^/kg, respectively (Fig. 2B). The particle size distribution of CD63-LMS ranged from 5.6 to 20.5 nm, with an average size of 12.62 ± 4.83 nm and a polydispersity index (PDI) of 0.107 (Fig. 2C). The average zeta potential of CD63-LMS was 10.35 ± 4.13 mv (Fig. 2D). AFM and TEM observations revealed that CD63-LMS exhibited a spherical shape with regular morphology, displaying vesicular features characteristic of liposomes (Fig. 2E and F). CD63-LMS showed an ultraviolet absorption peak at 276 nm (Fig. 2G). Infrared spectra of CD63-LMS indicated characteristic absorption peaks at 1714.1 cm^− 1^ (C = O stretching vibration of ester), 1112.7 cm^− 1^ (C-O-C stretching vibration of ester bond), 2849.55 cm^− 1^ (-CH- stretching vibration), and 1652.8 cm^− 1^ (C = O-NH stretching vibration of amide bond) (Fig. 2H).

### Identification of exosome

**Fig. 3:**
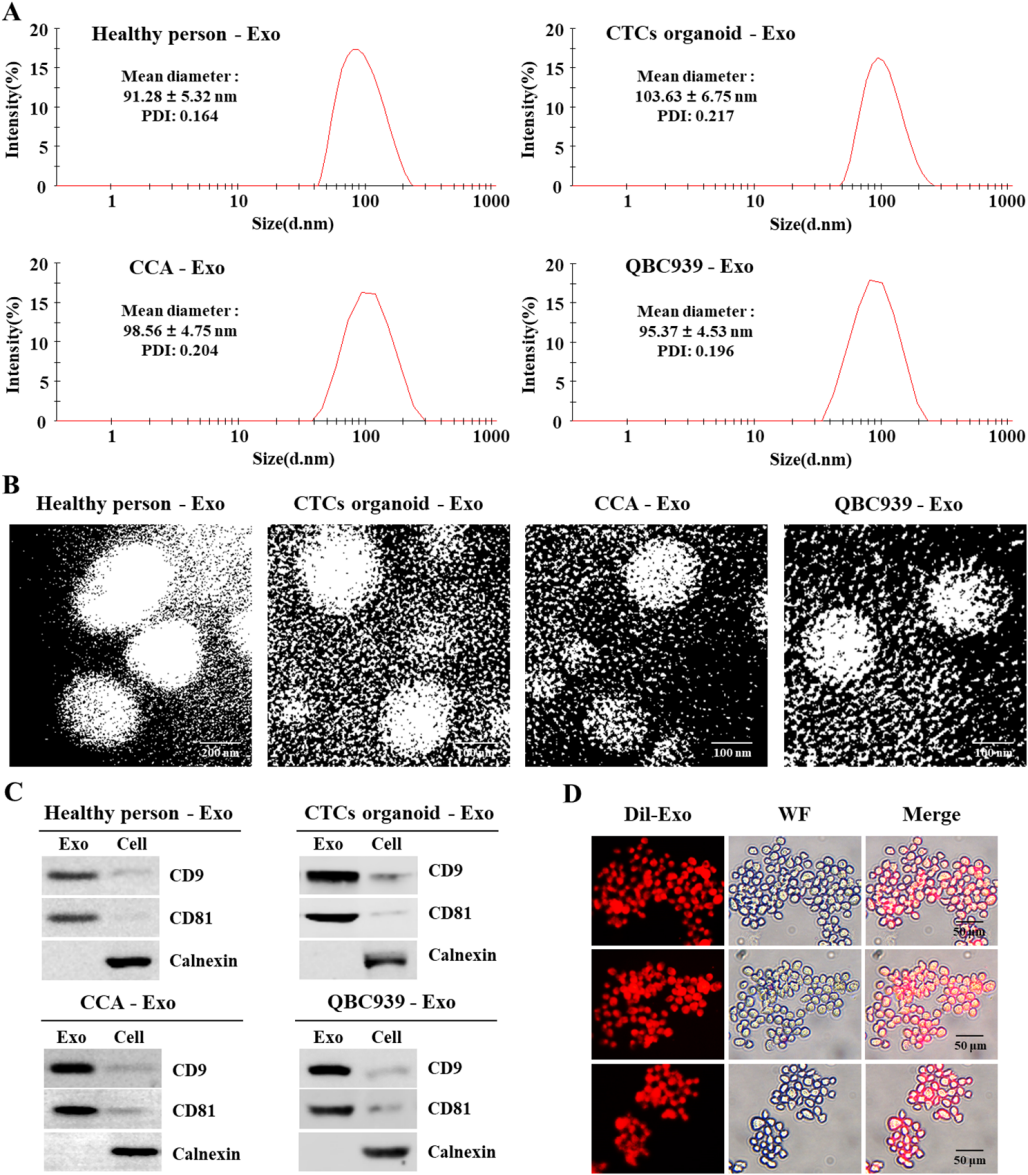
Identification of Exosomes: (**A**) Particle size distribution of exosomes; (**B**) TEM observation of spherical exosomes; (**C**) Western blot analysis of CD9, CD81, and Calnexin in exosomes; (**D**) Uptake of Dil-labeled exosomes by Huh28 cells

The average particle sizes of exosomes from healthy individuals’ blood, cholangiocarcinoma patients’ blood, CTCs organoid-derived exosomes, and QBC939 cell-derived exosomes were 99.28 ± 5.32 nm, 98.56 ± 4.75 nm, 103.63 ± 6.75 nm, and 95.37 ± 4.53 nm, respectively. The polydispersity indices (PDIs) were 0.164, 0.204, 0.217, and 0.196, respectively (Fig. 3A). Transmission electron microscopy revealed captured exosomes as nanoscale, quasi-circular membranous vesicles (Fig. 3B). The captured exosomes contained exosomal markers CD9 and CD81, while lacking the negative marker calnexin (Fig. 3C). Flow cytometry imaging analysis displayed labeled exosomes around the cytoplasm and nucleus of Huh28 cells, indicating effective uptake of exosomes by Huh28 cells (Fig. 3D).

### Identification and screening of LncRNA in exosomes secreted by cholangiocarcinoma tumor cells

**Fig. 4:**
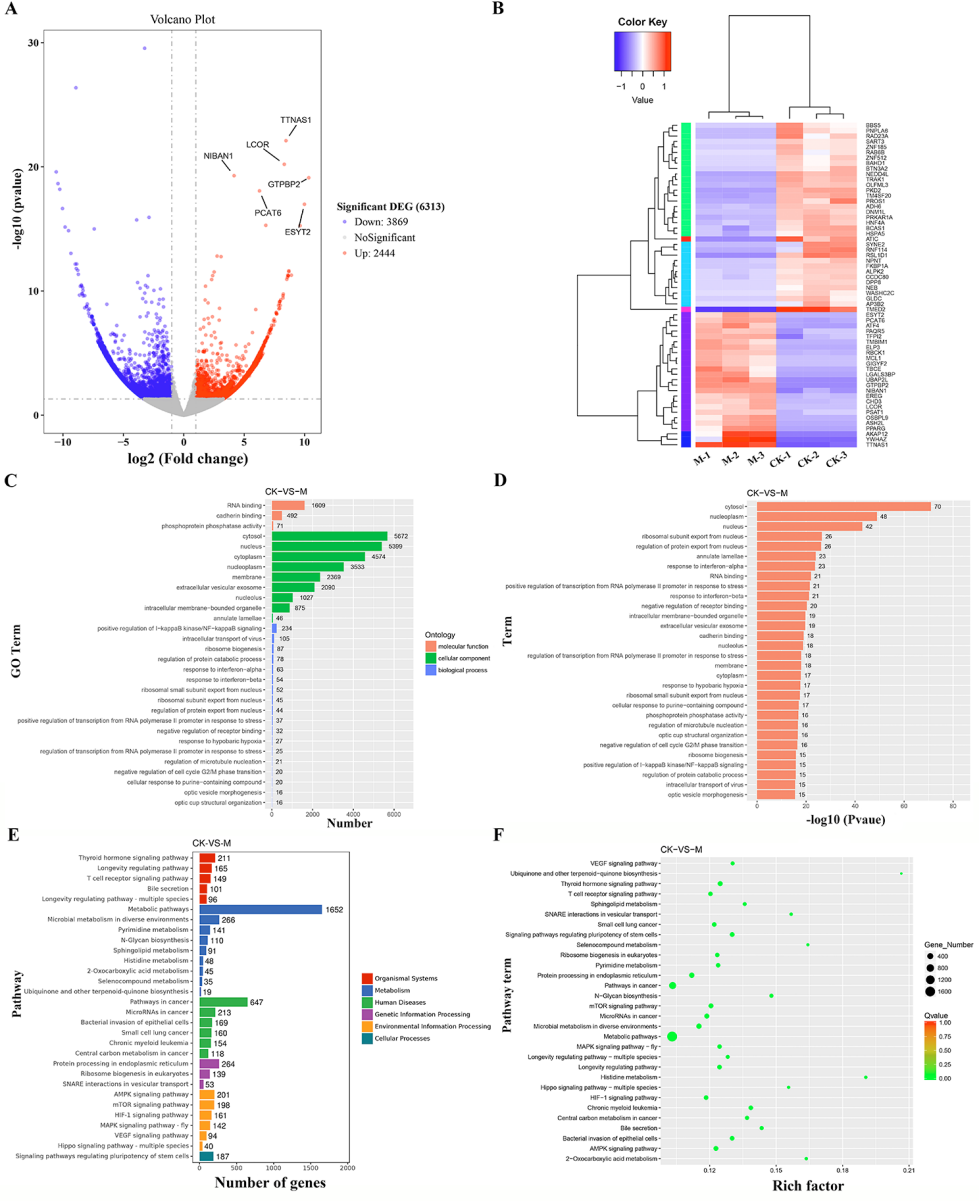
Screening and Functional Analysis of LncRNAs. (**A**) Volcano plot of differentially expressed LncRNAs between M group (*n* = 3) and CK group (*n* = 3) samples. Red and blue dots represent upregulated and downregulated genes, respectively, while gray dots indicate genes without statistical significance; (**B**) Hierarchical clustering analysis of differentially expressed LncRNAs, with red indicating high expression and blue indicating low expression. Color gradient from blue to red represents increasing LncRNA expression levels; (**C**) Distribution of differentially expressed genes in the top 30 enriched GO terms across Biological Process, Cellular Component, and Molecular Function categories; (**D**) Bar graph showing the P-values of enriched GO terms; (**E**) Bar chart illustrating significantly enriched KEGG pathway annotations; (**F**) Scatter plot displaying enriched KEGG pathways for differentially expressed genes

The identification and prediction process of LncRNAs was carried out for the transcriptome sequencing analysis of extracellular vesicles secreted by cholangiocarcinoma tumor cells and normal cells (Supplementary Figure S2A). A total of 33,731 candidate LncRNAs were selected using the Pfam database in combination with CPC and CNCI software (Supplementary Figure S2B). The number of known and unknown LncRNA were counted and summarized using authoritative databases (Supplementary Figure S2C), the length of the screened LncRNA (Supplementary Figure S2D) and the number of exons (Supplementary Figure S2E) were counted and classified (Supplementary Figure S2F). Additionally, the distribution and box plots of FPKM for all LncRNAs were used to compare LncRNA expression levels under different experimental conditions (Supplementary Figure S2G-H). After applying stringent differential expression criteria, 3,869 downregulated and 2,444 upregulated LncRNAs were identified (Supplementary Figure S2I). Among them, LncRNAs such as TTN-AS1, LCOR, NIBAN1, GTPBP2, PCAT6, and ESYT2 showed significant upregulation. Cluster analysis revealed that TTN-AS1 exhibited consistently higher expression levels in extracellular vesicles secreted by cholangiocarcinoma tumor cells across 3 cases (Fig. 4A-B). Subsequently, the most significantly enriched 30 GO terms and pathways were selected for functional analysis of differentially expressed LncRNA target genes (Supplementary document 7). Notably, GO terms enriched by cellular component such as cytosol showed the highest differentially expressed genes (Fig. 4C-D). Among the pathways, metabolic pathways and pathways in cancer contained the highest number of differentially expressed genes (Fig. 4E-F). These analyses collectively suggest substantial differences in LncRNAs secreted by cholangiocarcinoma tumor cells and normal cells. Genes such as TTN-AS1, which exhibited notably elevated expression in extracellular vesicles secreted by cholangiocarcinoma tumor cells, hold promise as potential biomarkers for cholangiocarcinoma diagnosis and treatment.

### Diagnostic value of CCA tumor tissue, blood, and serum exosomes LncRNA TTN-AS1

**Fig. 5:**
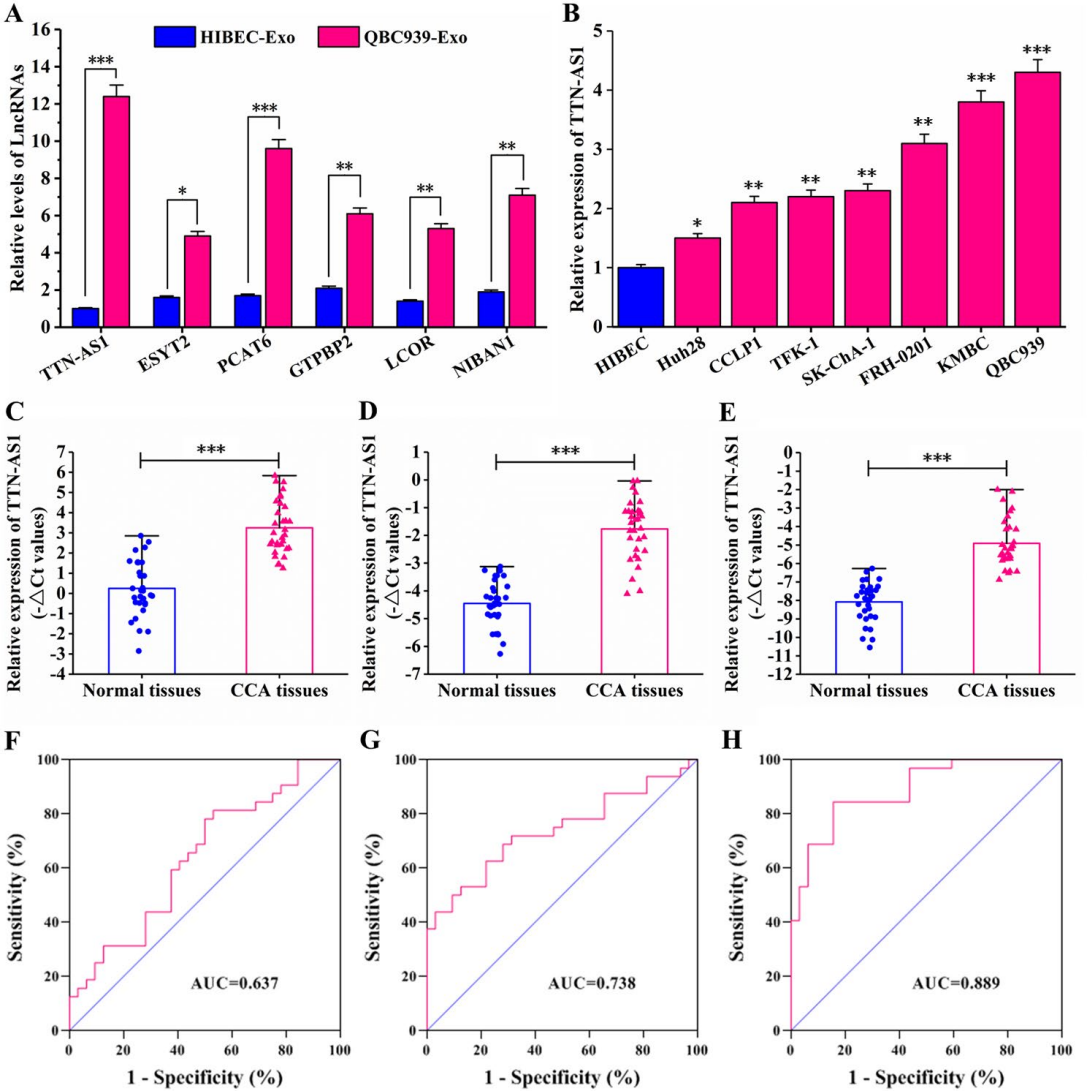
Expression of LncRNA TTN-AS1 in CCA cell lines, tissues, serum, and serum exosomes. (**A**) Detection of LncRNA expression in QBC939 and HIBEC cell-derived exosomes by qRT-PCR; (**B**) Detection of TTN-AS1 expression in cholangiocarcinoma cell lines by qRT-PCR; (**C**) Detection of TTN-AS1 expression in cholangiocarcinoma tissues and adjacent tissues by qRT-PCR; (**D**) Detection of TTN-AS1 expression in blood samples of cholangiocarcinoma patients and healthy individuals by qRT-PCR; (**E**) Detection of TTN-AS1 expression in serum exosomes of cholangiocarcinoma patients and healthy individuals by qRT-PCR; (**F**) ROC curve of TTN-AS1 expression in cholangiocarcinoma tumor tissues of patients; (**G**) ROC curve of TTN-AS1 expression in blood samples of cholangiocarcinoma patients; (**H**) ROC curve of TTN-AS1 expression in serum exosomes of cholangiocarcinoma patients

To investigate the clinical significance of TTN-AS1 in GC, we examined the expression levels of TTN-AS1 in tumor tissues of CCA patients, as well as in the blood, serum exosomes, and in CCA cell lines (Fig. 8A). The results indicated that among the 6 significantly upregulated genes, TTN-AS1 exhibited the most significant difference between exosomes derived from QBC939 and HIBEC cells (Fig. 5A). Additionally, TTN-AS1 was found to be downregulated in Huh28 and upregulated in QBC939 (Fig. 5B). The qRT-PCR results showed that TTN-AS1 was highly expressed in cholangiocarcinoma tissues (Fig. 5C, Supplementary Figure S3). The expression of TTN-AS1 in the blood and serum exosomes of cholangiocarcinoma patients was significantly higher compared to healthy individuals (Fig. 5D-E). Furthermore, using the expression of TTN-AS1 in normal tissues as a control, the ROC curve of TTN-AS1 in cholangiocarcinoma tissues was plotted, resulting in an AUC of 0.637 with a 95% confidence interval of 0.501–0.773, a sensitivity of 68.75%, and a specificity of 69.13% (Fig. 5F). Using the expression of TTN-AS1 in healthy individuals’ blood as a control, the ROC curve of TTN-AS1 in cholangiocarcinoma patients’ blood was plotted, resulting in an AUC of 0.738 with a 95% confidence interval of 0.614–0.863, a sensitivity of 71.88%, and a specificity of 68.75% (Fig. 5G). Using the expression of TTN-AS1 in healthy individuals’ serum exosomes as a control, the ROC curve of TTN-AS1 in cholangiocarcinoma patients’ serum exosomes was plotted, resulting in an AUC of 0.889 with a 95% confidence interval of 0.811–0.966, a sensitivity of 84.4%, and a specificity of 84.4% (Fig. 5H). Clinical correlation analysis revealed that the expression of LncRNA TTN-AS1 in cholangiocarcinoma patients’ tumor tissues was associated with lymph node metastasis (*P* = 0.032) and TNM stage (*P* = 0.008) (Supplementary Table S3). The expression of LncRNA TTN-AS1 in cholangiocarcinoma patients’ blood was correlated with lymph node metastasis (*P* = 0.009) and TNM stage (*P* = 0.018) (Supplementary Table S4). The expression of TTN-AS1 in cholangiocarcinoma patients’ serum exosomes was significantly correlated with lymph node metastasis (*P* = 0.005) and TNM stage (*P* = 0.003) (Table 1).

**Table 1:** Clinical correlation analysis of LncRNA TTN-AS1 expression in serum extracellular vesicles of patients with cholangiocarcinoma

Features	Number	Mean ± SD	P value	Significance
Gender				
Male	19	-5.34 ± 1.95	0.684	
Female	13	-4.64 ± 2.34		
Age (years)				
< 60	20	-4.94 ± 2.81	0.314	
≥ 60	12	-5.34 ± 1.96		
Tumor size				
< 5 cm	17	-5.64 ± 2.84	0.297	
≥ 5 cm	15	-4.71 ± 3.41		
Metastasis				
Negative	12	-5.13 ± 2.64	0.005	**
Positive	20	-6.71 ± 1.18		
Tumor differentiation				
Well/moderate	18	-5.84 ± 2.14	0.378	
Poor	14	-5.17 ± 1.98		
Portal vein invasion				
No	15	-5.67 ± 1.94	0.648	
Yes	17	-4.84 ± 2.38		
Tumor location				
Intrahepatic	10	-5.84 ± 2.67	0.651	
Perihilar	22	-4.25 ± 1.49		
TNM stage				
I/II	15	-4.75 ± 2.19	0.003	**
III / IV	17	-6.18 ± 1.67		

### CTCs derived exosome TTN-AS1 can promote the proliferation and migration of cholangiocarcinoma cells

**Fig. 6:**
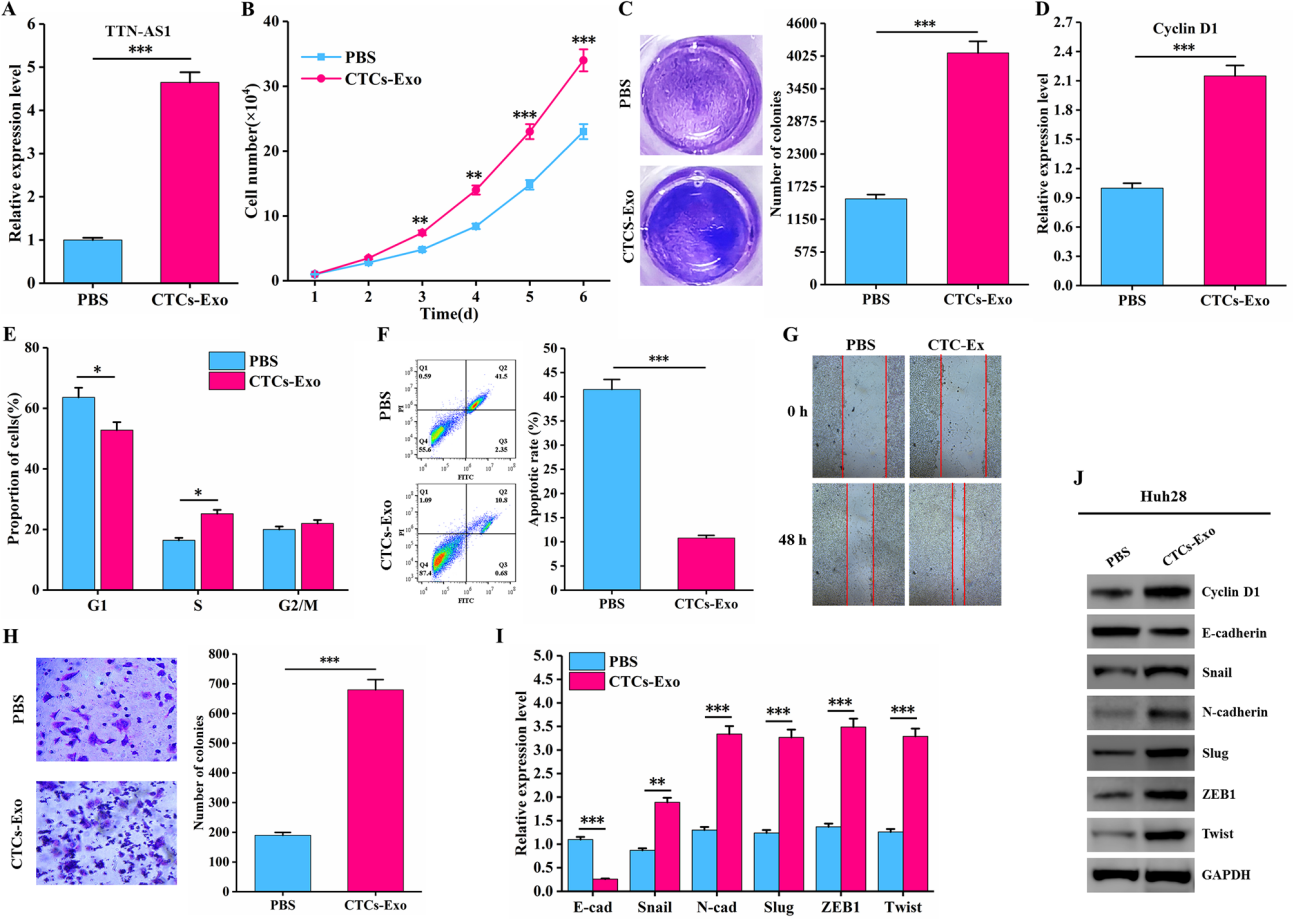
The effect of exosomes secreted by cholangiocarcinoma CTCs-organoids on the proliferation and migration of Huh28 cells. (**A**) Significant upregulation of TTN-AS1 expression in Huh28 cells co-cultured with CTCs-exosomes; (**B**) CTCs-exosomes promote the growth of Huh28 cells; (**C**) CTCs-exosomes enhance the clonogenicity of Huh28 cells; (**D**) CTCs-exosomes increase Cyclin D1 mRNA expression in Huh28 cells; (**E**) CTCs-exosomes accelerate the cell cycle progression of Huh28 cells; (**F**) CTCs-exosomes inhibit apoptosis of Huh28 cells; (**G**) CTCs-exosomes facilitate the migration of Huh28 cells; (**H**) CTCs-exosomes promote the migration of Huh28 cells; (**I**) Expression of EMT-related genes in Huh28 cells after co-culture with CTCs-exosomes; (**J**) Expression of Cyclin D1 and EMT-related proteins in Huh28 cells after co-culture with CTCs-exosomes

**Fig. 7:**
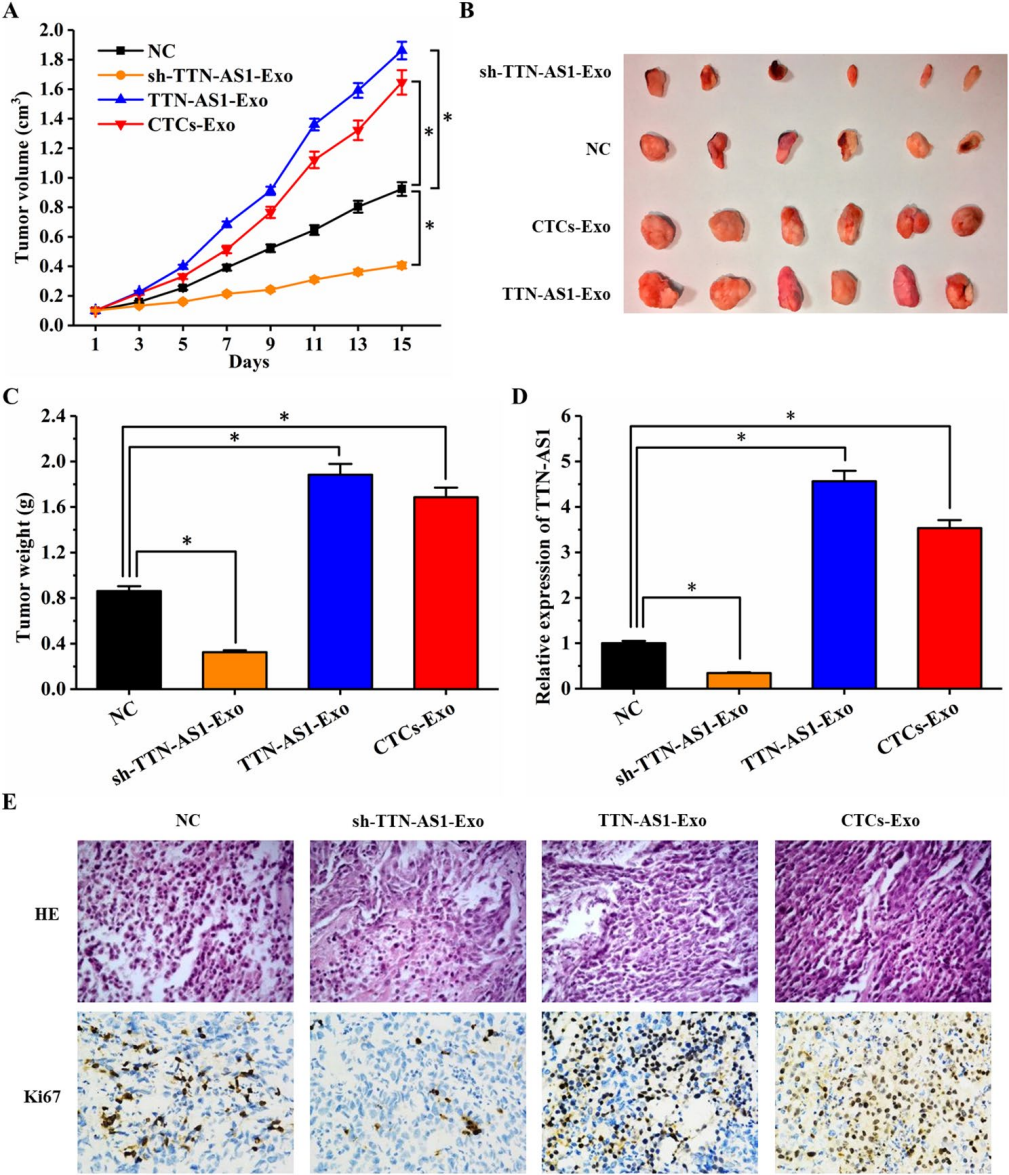
Effects of LncRNA TTN-AS1 depletion, overexpression, and CTC-derived exosomes on tumor growth in vivo. (**A**) Measurement of tumor volume changes every day; (**B**) Photographs of excised tumors; (**C**) Average weight of excised tumors; (**D**) Expression of TTN-AS1 in tumor tissues of each group detected by qRT-PCR; (**E**) H&E staining and immunohistochemical analysis of tumor tissues in each experimental group

Exosomes are considered important mediators of information transfer between donor and recipient cells. Therefore, we treated Huh28 cells with CCA CTCs-exosomes for 24 h to investigate whether the transfer of TTN-AS1 mediated by CTCs-exosomes would affect the behavior of target cells. The results showed that co-culturing CTCs-exosomes with Huh28 cells led to a significant increase in TTN-AS1 expression (Fig. 6A), a noticeable increase in cell growth (Fig. 6B), and a significant increase in the number of colony-forming units of Huh28 cells (Fig. 6C). After co-culture, both mRNA and protein expression levels of cell cycle protein Cyclin D1 significantly increased (Fig. 6D and J), resulting in a reduction of cells in the G1 phase and a significant increase in cells in the S phase (Fig. 6E). Co-culture with CTCs-exosomes led to a significant reduction in cell apoptosis rate (Fig. 6F). Enhanced cell migration ability was observed after co-culturing with CTCs-exosomes (Fig. 6G and H). Following co-culture, E-cadherin mRNA and protein expression levels significantly decreased, while N-cadherin, Slug, Snail, Twist, and ZEB1 mRNA and protein expression levels significantly increased (Fig. 6I and J). This indicates that cholangiocarcinoma CTCs are able to secrete exosomes carrying LncRNA TTN-AS1, promoting cholangiocarcinoma metastasis (Fig. 7B).

### Exosomes with high expression of TTN-AS1 can promote the proliferation and migration of cholangiocarcinoma cells

To further demonstrate the functional role of TTN-AS1 in CCA, we overexpressed TTN-AS1 in Huh28 cells. The results showed that the expression level of TTN-AS1 was significantly increased in transfected cells (Supplementary Figure S4A). After co-culturing Huh28 cells with CTCs-exosomes overexpressing TTN-AS1, there was a significant increase in TTN-AS1 expression (Supplementary Figure S5A), cell growth was notably enhanced (Supplementary Figure S5B), and the number of colony-forming units significantly increased (Supplementary Figure S5C). Upon co-culture, mRNA and protein expression levels of cell cycle protein Cyclin D1 significantly increased (Supplementary Figure S5D, Supplementary Figure S5J), resulting in a decrease in G1-phase cells and a notable increase in S-phase cells (Supplementary Figure S5E). Co-culture with CTCs-exosomes overexpressing TTN-AS1 led to a significant reduction in cell apoptosis rate (Supplementary Figure S5F). Enhanced cell migration ability was observed after co-culturing with CTCs-exosomes overexpressing TTN-AS1 (Supplementary Figure S5G, Supplementary Figure S5H). Furthermore, mRNA and protein expression levels of E-cadherin were significantly decreased, while N-cadherin, Slug, Snail, Twist, and ZEB1 mRNA and protein expression levels were significantly increased after co-culture (Supplementary Figure S5I, Supplementary Figure S5J).

### Exosomes with low expression of TTN-AS1 can inhibit the proliferation and migration of cholangiocarcinoma cells

To further validate the promotive role of TTN-AS1 in CCA, we knocked down TTN-AS1 in QBC-939 cells. The results showed that the expression level of TTN-AS1 was significantly decreased in transfected cells (Supplementary Figure S4B). After co-culturing QBC-939 cells with CTCs-exosomes with low TTN-AS1 expression, there was a significant reduction in TTN-AS1 expression (Supplementary Figure S6A), and QBC-939 cell growth significantly decreased (Supplementary Figure S6B), along with a notable decrease in the number of colony-forming units (Supplementary Figure S6C). Upon co-culture, mRNA and protein expression levels of cell cycle protein Cyclin D1 significantly decreased (Supplementary Figure S6D, Supplementary Figure S6J), leading to an increase in G1-phase cells and a notable decrease in S-phase cells (Supplementary Figure S6E). Co-culture with CTCs-exosomes with low TTN-AS1 expression resulted in a significant increase in cell apoptosis rate (Supplementary Figure S6F). The migration ability of QBC-939 cells was notably weakened after co-culturing with CTCs-exosomes with low TTN-AS1 expression (Supplementary Figure S6G, Supplementary Figure S6H). Moreover, mRNA and protein expression levels of E-cadherin were significantly increased, while N-cadherin, Slug, Snail, Twist, and ZEB1 mRNA and protein expression levels were significantly decreased after co-culture (Supplementary Figure S6I, Supplementary Figure S6J).

### Effects of TTN-AS1 deficiency, overexpression, and CTC-derived exosome on in vivo tumors

To further explore the effects of TTN-AS1 depletion, overexpression, and CTC-derived exosomes containing PTENP1 in CCA in vivo, sh-TTN-AS1-Exo, TTN-AS1-Exo, and CTCs-Exo were co-cultured with Huh28 cells and then injected into nude mice. The results showed that compared to the NC group, overexpression of TTN-AS1 and CTC-derived exosomes co-cultured with Huh28 cells significantly increased the average tumor volume and average tumor weight, while TTN-AS1-depleted exosomes co-cultured with Huh28 cells significantly reduced the average tumor volume and average tumor weight (Fig. 7A-C). After co-culture with TTN-AS1 overexpressing and CTC-derived exosomes, TTN-AS1 expression increased, while co-culture with TTN-AS1-depleted exosomes led to decreased TTN-AS1 expression (Fig. 7D). Additionally, H&E staining and immunohistochemistry were performed on proliferation marker Ki67. The results showed that after co-culture with TTN-AS1 overexpressing and CTC-derived exosomes, Ki67 expression was significantly increased, whereas co-culture with TTN-AS1-depleted exosomes resulted in a significant decrease in Ki67 expression (Fig. 7E).

**Fig. 8:**
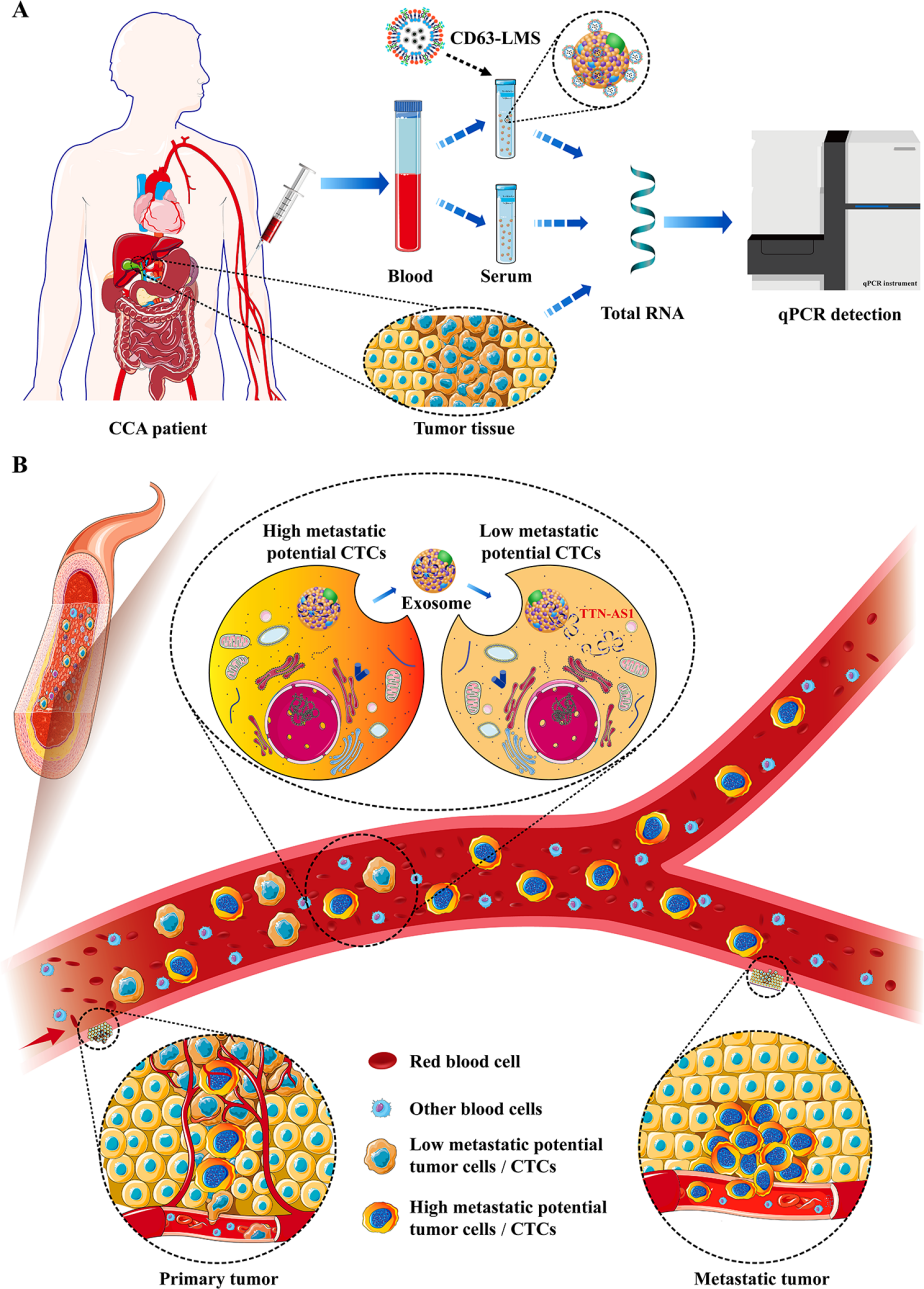
Detection of LncRNA TTN-AS1 and schematic representation of its promotion of cholangiocarcinoma metastasis. (**A**) Flowchart for the detection of cholangiocarcinoma LncRNA TTN-AS1; (**B**) Schematic representation of cholangiocarcinoma CTC-derived exosomes carrying LncRNA TTN-AS1 promoting cholangiocarcinoma metastasis

## Discussion

The primary curative method for cholangiocarcinoma is surgical resection of the primary tumor, but only 26% of patients are eligible for surgery. Due to occult metastasis occurring in some patients at the time of surgery, the 5-year survival rate is quite low [26–28]. Therefore, exploring the molecular biology mechanisms of cholangiocarcinoma initiation and progression, as well as effective molecular markers, is essential. Circulating tumor cells (CTCs) shed into the bloodstream from the primary tumor or distant metastatic lesions are considered as precursors of metastasis, playing a crucial role in tumor dissemination and the metastatic process [2]. However, most CTCs do not travel as isolated single cells in the blood, but rather accompany a large number of blood cells [29, 30]. Recent studies suggest that the interaction and modulation of CTCs with the blood microenvironment are critical for the development of new metastases, and interfering with this new microenvironment might help reduce the potential for tumor dissemination [31]. Exosomes, as a part of the microenvironment, have been discovered in recent years to play a key role in intercellular communication between cells and are essential components of extracellular matrix. Tumor-derived exosomes interact with stromal cells (including tumor cells) in the tumor microenvironment and can promote tumor cell metastasis [32–34]. Therefore, this study established a cholangiocarcinoma CTCs organoid model and, for the first time, performed transcriptome sequencing on exosomes secreted by cholangiocarcinoma CTCs organoids. The study discovered that TTN-AS1 is significantly upregulated in exosomes derived from cholangiocarcinoma CTCs, and this was further validated at both the cellular and animal levels. The findings confirmed that exosomes from CCA CTCs could transport TTN-AS1 to promote the growth and metastasis of CCA cells.

Aberrant expression of long non-coding RNAs (LncRNAs) plays a crucial role in various cancers. For instance, LncRNA PCAT6 is upregulated in cholangiocarcinoma tissues compared to normal tissues, and its expression is correlated with TNM staging [35]. LncRNA MT1JP is downregulated in cholangiocarcinoma tissues, and its expression is associated with TNM staging and lymph node metastasis [36]. LncRNA TTN-AS1 is highly expressed in cholangiocarcinoma tissues [37]. Moreover, exosomal delivery of TTN-AS1 derived from gastric cancer cells can promote gastric cancer progression [38]. Elevated levels of exosomal LncRNA TTN-AS1 expression have been observed in bladder cancer and have diagnostic value [39]. In our previous studies, we identified high expression of LncRNA TTN-AS1 in cholangiocarcinoma tissues. Its expression was correlated with portal vein invasion, tumor differentiation, and lymph node metastasis [25]. This current study also confirmed the high expression of LncRNA TTN-AS1 in cholangiocarcinoma tissues and found a significant association between TTN-AS1 expression and lymph node metastasis and TNM staging. Furthermore, this research revealed elevated expression of LncRNA TTN-AS1 in the blood and serum exosomes of cholangiocarcinoma patients, both of which were correlated with TNM staging and lymph node metastasis. Notably, the area under the curve (AUC) for TTN-AS1 expression in serum exosomes was higher than that in tumor tissues and blood, with an AUC of 0.889, a 95% confidence interval of 0.811–0.966, sensitivity of 84.4%, and specificity of 84.4%. This suggests that TTN-AS1 in serum exosomes holds promise as a potential biomarker for cholangiocarcinoma diagnosis and treatment. The delivery of TTN-AS1 through exosomes could provide a new avenue for cholangiocarcinoma therapy and offers experimental evidence for investigating the biological functions of exosomal LncRNA TTN-AS1 secreted by cholangiocarcinoma CTCs.

This study provides the first evidence that cholangiocarcinoma patients’ serum exosomes contain abundant LncRNA TTN-AS1. The diagnostic and predictive value of LncRNA TTN-AS1 in serum exosomes surpasses that of tumor tissues and blood. However, whether the highly expressed TTN-AS1 originates from tumor cell-secreted exosomes remains unclear. Furthermore, to confirm whether tumor cells indeed secrete exosomes rich in TTN-AS1 and whether they interact with stromal cells (including tumor cells) in the tumor microenvironment to promote cholangiocarcinoma metastasis, this research established a cholangiocarcinoma CTCs organoid model, overexpressed and silenced TTN-AS1 in cholangiocarcinoma cell lines and co-cultured the captured exosomes with cholangiocarcinoma cell lines with low TTN-AS1 expression. This approach was pursued both at the cellular and animal levels. The findings confirmed that cholangiocarcinoma CTCs can secrete exosomes carrying TTN-AS1, thereby promoting cholangiocarcinoma metastasis (Fig. 7B). The molecular mechanisms through which TTN-AS1 promotes cholangiocarcinoma progression have been elucidated in our prior research [25]. However, whether it mediates its effects on cholangiocarcinoma progression through exosomes requires further investigation to be confirmed. Addressing this question would provide a comprehensive understanding of the role of TTN-AS1 in exosomes secreted by cholangiocarcinoma tumor cells and hold significant guiding implications for utilizing exosomal LncRNA TTN-AS1 as a novel target for cholangiocarcinoma diagnosis and treatment.

## Conclusions

This study has revealed that the diagnostic and predictive value of LncRNA TTN-AS1 in serum exosomes surpasses that of tissue and blood. Exosomes derived from cholangiocarcinoma CTCs have the capability to transport LncRNA TTN-AS1 and promote cholangiocarcinoma metastasis. These findings strongly suggest that exosomal LncRNA TTN-AS1 sourced from CTCs could serve as a potential biomarker for cholangiocarcinoma diagnosis and treatment. The transfer of TTN-AS1 through exosomes from CTCs could open up new avenues for therapeutic strategies targeting cholangiocarcinoma.

### Electronic supplementary material

Below is the link to the electronic supplementary material.

Supplementary Material 1

### Electronic supplementary material

Below is the link to the electronic supplementary material.

Supplementary Material 2

### Electronic supplementary material

Below is the link to the electronic supplementary material.

Supplementary Material 3

### Electronic supplementary material

Below is the link to the electronic supplementary material.

Supplementary Material 4

### Electronic supplementary material

Below is the link to the electronic supplementary material.

Supplementary Material 5

### Electronic supplementary material

Below is the link to the electronic supplementary material.

Supplementary Material 6

### Electronic supplementary material

Below is the link to the electronic supplementary material.

Supplementary Material 7

## Data Availability

All data generated or analysed during this study are included in this published article [and its supplementary information files].
