# Supplementary figures and images for: Circulating tumor cell-derived exosome–transmitted long non-coding RNA TTN-AS1 can promote the proliferation and migration of cholangiocarcinoma cells
Source: J Nanobiotechnology. 2024 Apr 18;22:191. doi: 10.1186/s12951-024-02459-8 (PMC11025154; doi:10.1186/s12951-024-02459-8)

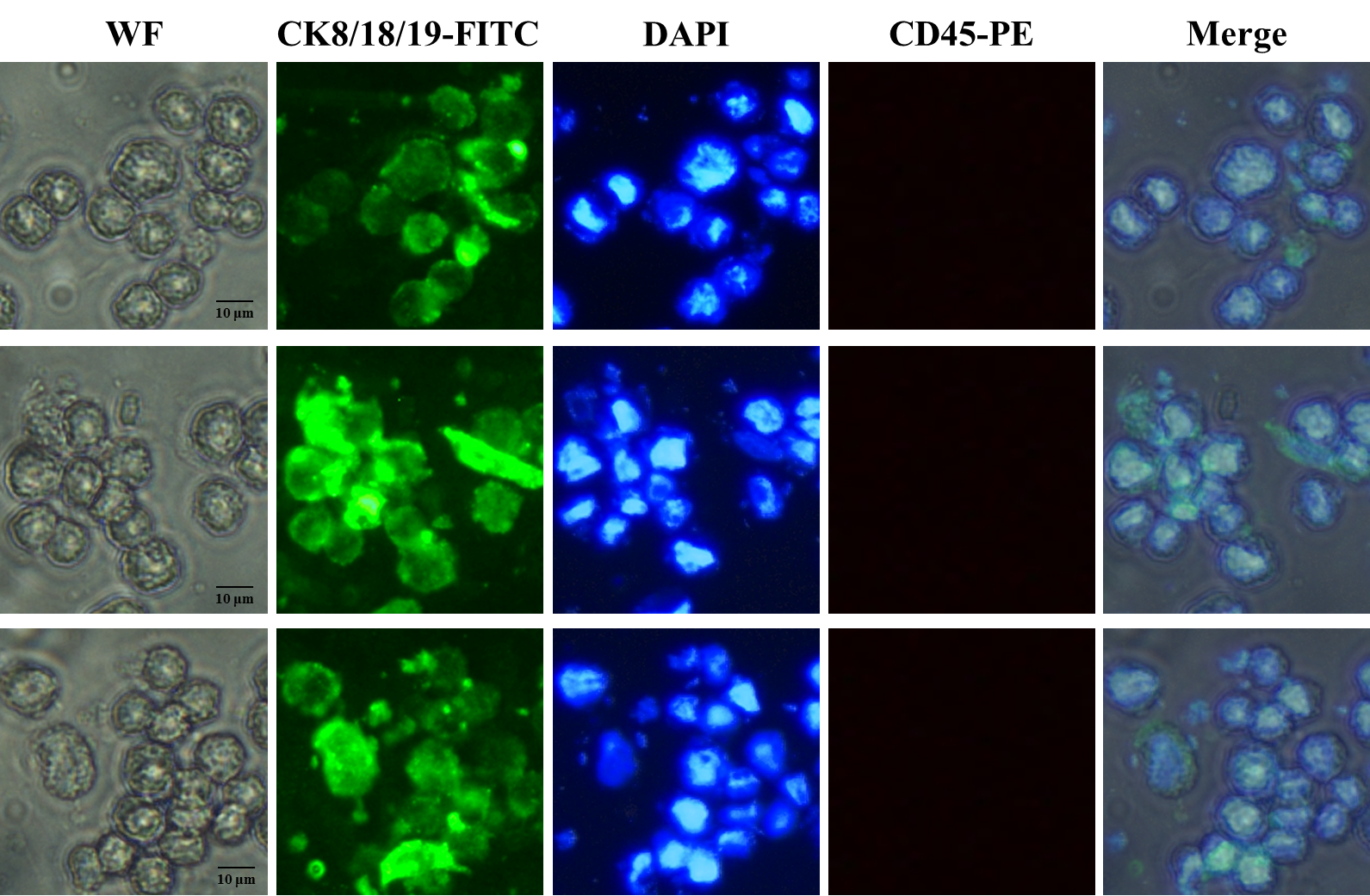

Supplement: Supplementary file 1 — Supplementary Material 1 [file 12951_2024_2459_MOESM1_ESM.tif]

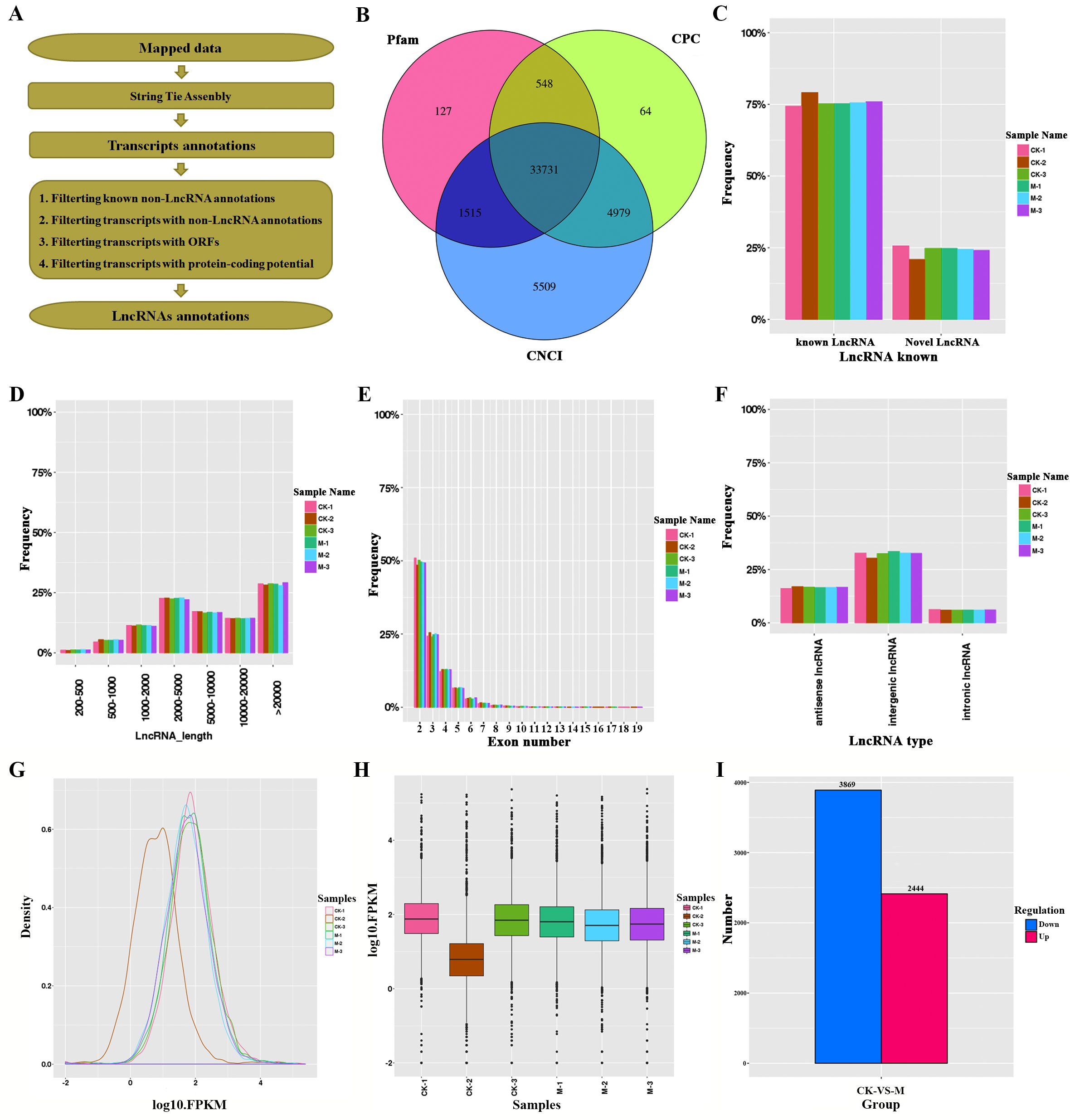

Supplement: Supplementary file 2 — Supplementary Material 2 [file 12951_2024_2459_MOESM2_ESM.tif]

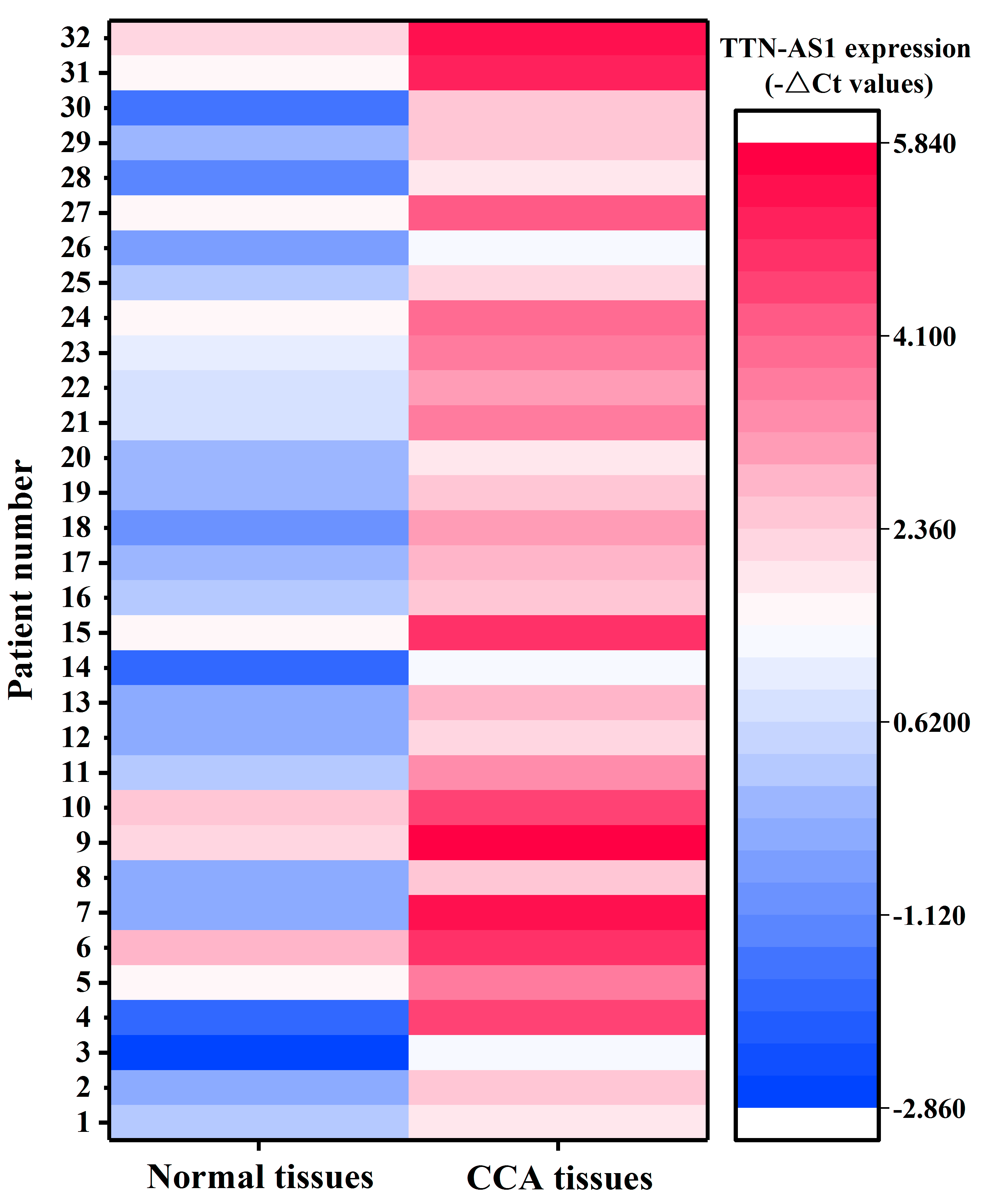

Supplement: Supplementary file 3 — Supplementary Material 3 [file 12951_2024_2459_MOESM3_ESM.tif]

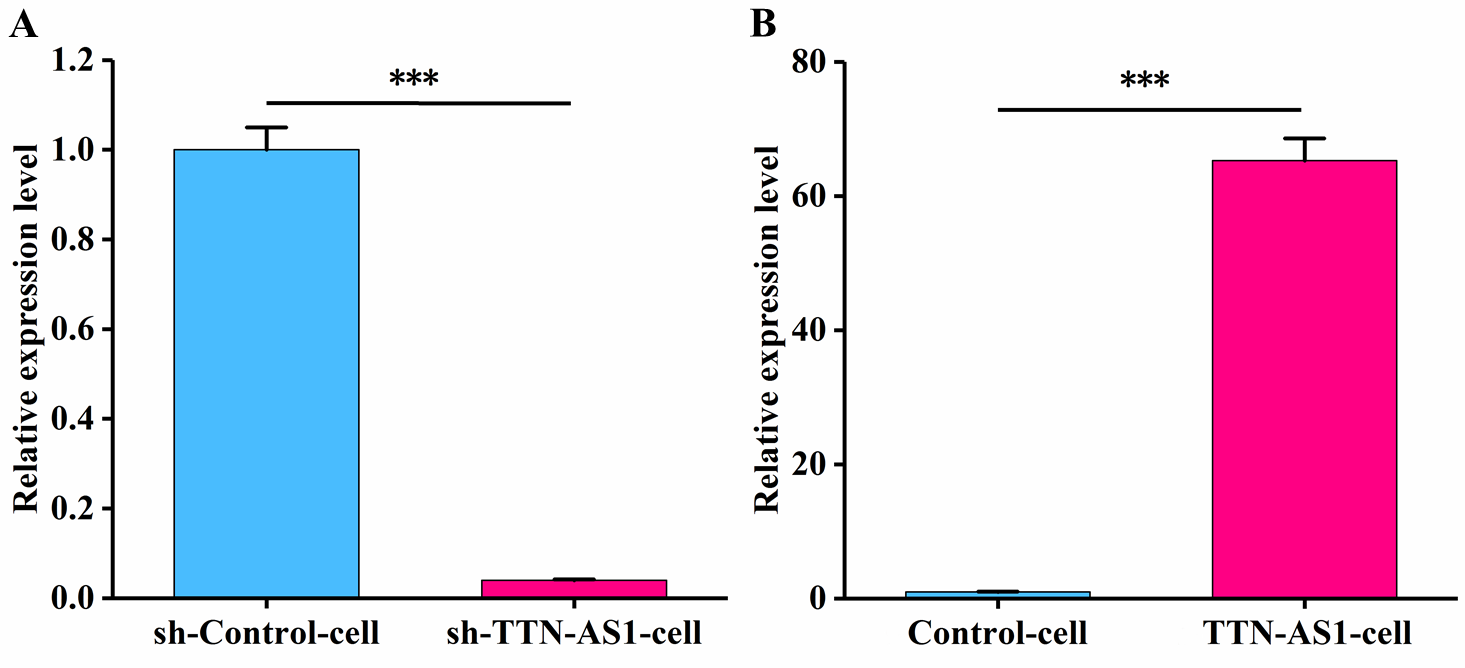

Supplement: Supplementary file 4 — Supplementary Material 4 [file 12951_2024_2459_MOESM4_ESM.tif]

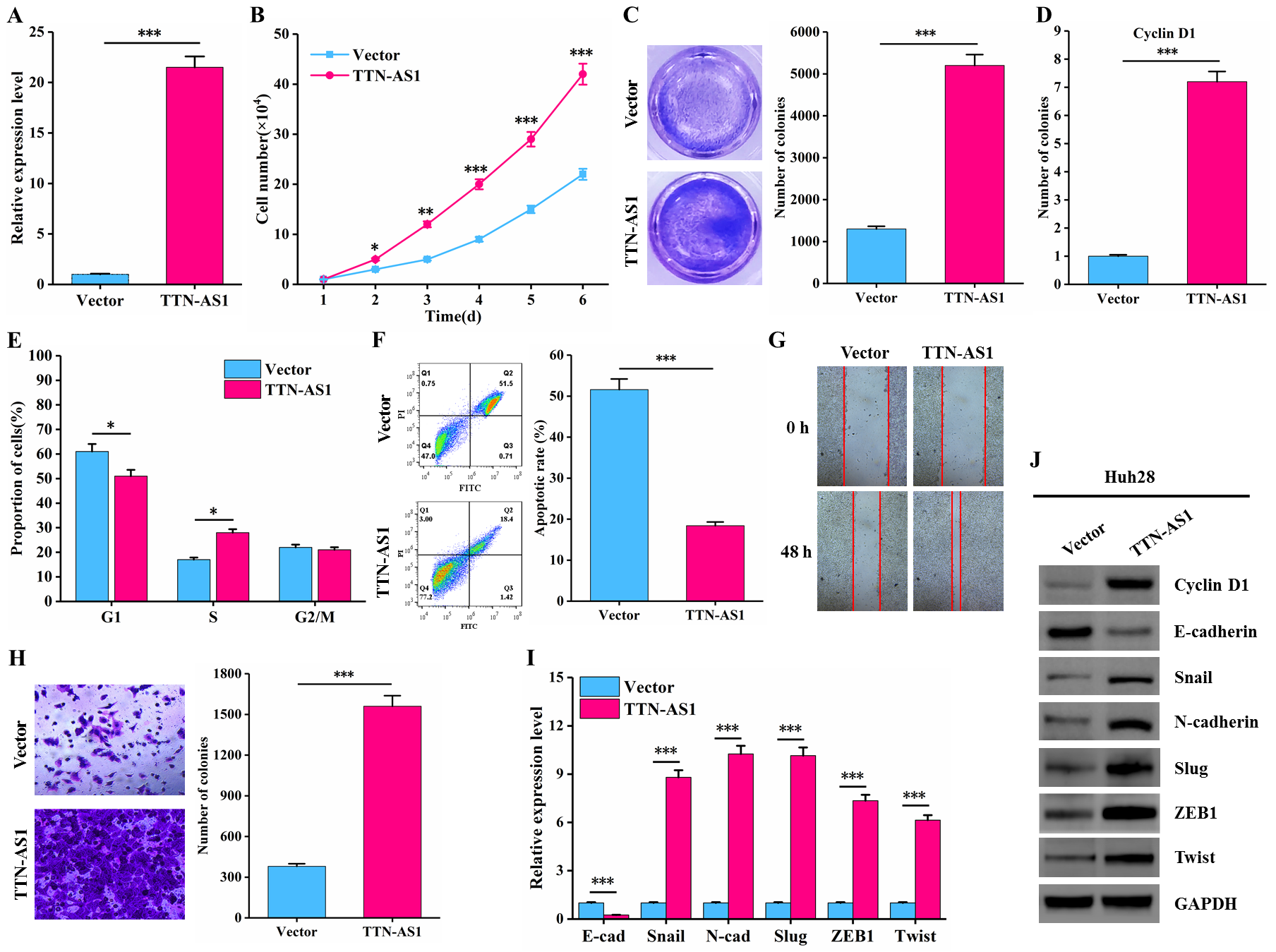

Supplement: Supplementary file 5 — Supplementary Material 5 [file 12951_2024_2459_MOESM5_ESM.tif]

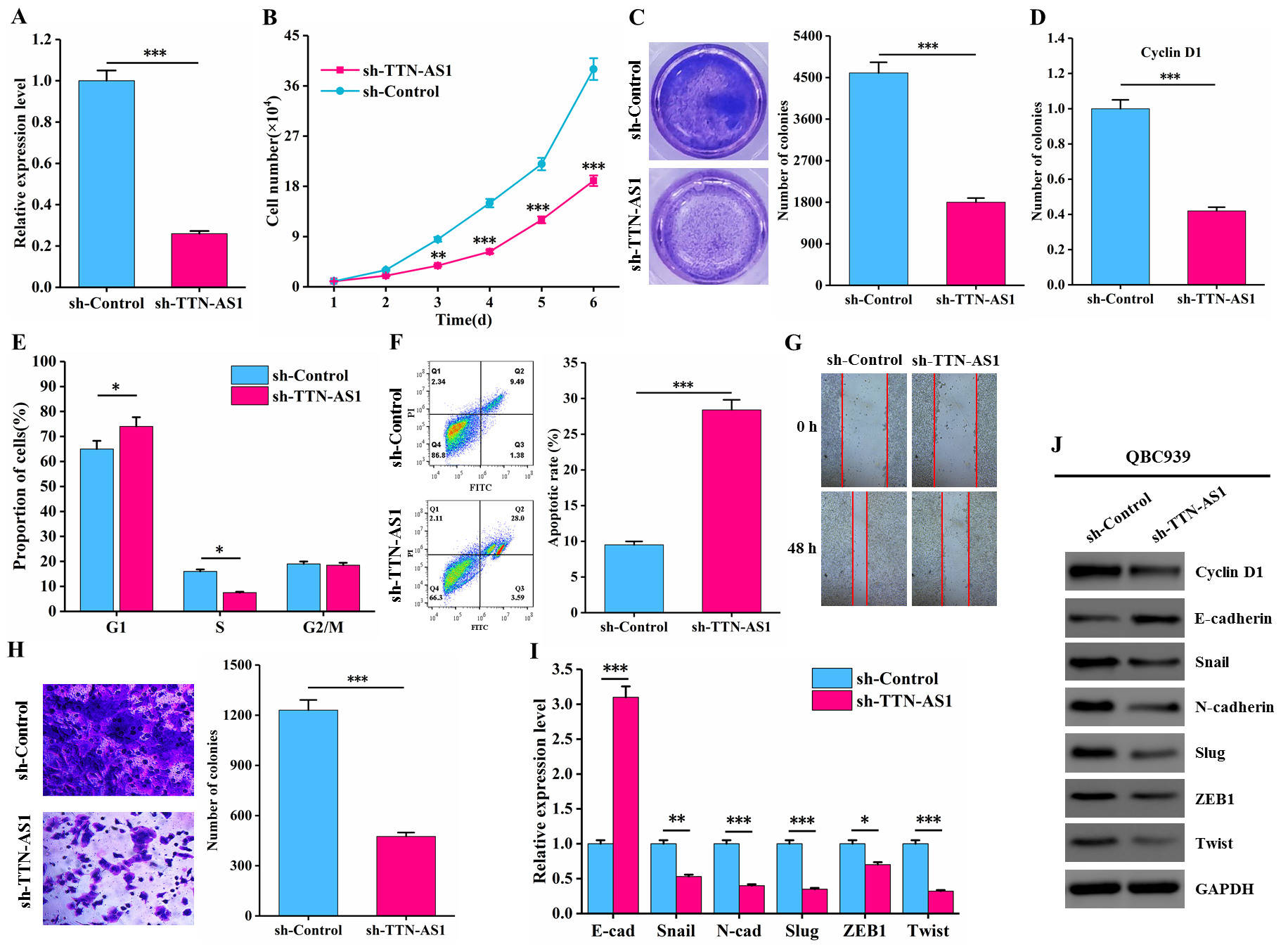

Supplement: Supplementary file 6 — Supplementary Material 6 [file 12951_2024_2459_MOESM6_ESM.tif]
